# Weight Loss Following Bariatric Surgery in People with or without Metabolic Syndrome: A 5-Year Observational Comparative Study

**DOI:** 10.3390/jcm13010256

**Published:** 2024-01-01

**Authors:** Sharanniyan Ragavan, Omar Elhelw, Waseem Majeed, Bilal Alkhaffaf, Siba Senapati, Basil J. Ammori, Rajshekhar N. Mudaliar, Akheel A. Syed

**Affiliations:** 1 School of Medical Sciences, The University of Manchester, Manchester M13 9PL, UK; sharanniyan.ragavan@nhs.net (S.R.);; 2 Department of Diabetes, Endocrinology and Obesity Medicine, Salford Royal Hospital, Northern Care Alliance NHS Foundation Trust, Salford M6 8HD, UK; 3 Faculty of Biology, Medicine and Health, The University of Manchester, Manchester M13 9PL, UK; 4 Department of Oesophago-Gastric and Bariatric Surgery, Salford Royal Hospital, Northern Care Alliance NHS Foundation Trust, Salford M6 8HD, UK; siba.senapati@nca.nhs.uk; 5 School of Health and Society, Allerton Concourse, Frederick Road Campus, University of Salford, Salford M6 6PU, UK; 6 Department of Bariatric, General, Gastrointestinal and Hepatobiliary Surgery, Burjeel Hospital, Al Najdah Street, Abu Dhabi P.O. Box 7400, United Arab Emirates

**Keywords:** metabolic syndrome, insulin resistance, gastric bypass, gastrectomy, weight loss

## Abstract

Aim: Whilst bariatric surgery is the most effective treatment for severe obesity, the aim of this study was to evaluate whether postoperative weight loss is similar in patients with or without metabolic syndrome. Methods: We performed a 5-year observational retrospective comparative cohort analysis of bariatric surgery in 333 patients (72% women) without (*Group A*, n = 133) or with (*Group B*, n = 200) metabolic syndrome at baseline. Results: Overall mean (SD) baseline body mass index was 51.7 (7.5) with no significant difference between groups. Overall mean percent total weight loss (%TWL) was 31.9% by 24 months after surgery. Although %TWL was greater in *Group A* (34.9%) than in *Group B* (30.2%, *p* = 0.006) at 24 months, there were no significant differences between groups subsequently up to 60 months of follow-up. Systolic and diastolic blood pressures and lipid profiles improved in both groups. In patients with metabolic syndrome at baseline, mean HbA1c reduced by 36.4% at 12 months and was sustained over the study period. Conclusions: We report that bariatric surgery results in comparable long-term weight loss in patients with or without metabolic syndrome alongside expected improvements in metabolic comorbidities.

## 1. Introduction

Obesity is a growing epidemic worldwide and will generate a continued health burden on the global population [1]. The prevalence of clinical obesity, commonly defined as a body mass index (BMI) ≥ 30.0 kg/m^2^, is higher in the United Kingdom than most other parts of northwest Europe [2]. The Foresight Report, commissioned in 2007, estimated the rate of obesity in the UK to have doubled in the previous 25 years with nearly 25% of adults living with obesity [3]. Data from the latest Health Survey for England estimate that 26% of adults in England are living with obesity and a further 38% are overweight (BMI ≥ 25.0 < 30.0 kg/m^2^) [4], including 69% of men and 59% of women living with excess weight (BMI ≥ 25.0 kg/m^2^).

Obesity increases the risk of developing metabolic comorbidities such as type 2 diabetes (T2D), hypertension, ischaemic heart disease, and obstructive sleep apnoea (OSA), as well as increased mortality in general [5]. Metabolic syndrome, originally described as ‘a cluster of risk factors for diabetes and cardiovascular disease’ [6], is associated with obesity as a key component, along with insulin resistance, dyslipidaemia and/or hypertension. While the definition of metabolic syndrome varies based on guidelines, having central obesity combined with two out of the following factors constitutes a diagnosis of metabolic syndrome: elevated triglycerides, reduced high-density lipoprotein (HDL) cholesterol, hypertension, and impaired glucose tolerance/T2D [7]. The presence of metabolic syndrome in individuals leads to a high risk of cardiovascular disease and death [8].

Treating obesity thus becomes a priority in decreasing the underlying obesity-related complications and the development of metabolic syndrome. Management of chronic obesity constitutes multi-disciplinary lifestyle, dietary and behavioural interventions, anti-obesity medications, and bariatric surgery [9]. There have been significant advances in medical therapies for obesity in recent times [10]. The approval of incretin-based therapies, including the glucagon-like peptide-1 (GLP1) receptor agonists Liraglutide [11] and Semaglutide [12], and the glucose-dependent insulinotropic polypeptide (GIP) and GLP1 dual agonist Tirzepatide [13], has brought new hope for the medical management of obesity. However, metabolic bariatric surgery remains the most effective evidence-based weight reduction intervention for severe chronic obesity [14].

In the UK, weight management is organised in tiers of interventions [15]. Whilst Tier 1 covers universal health promotion, Tier 2 includes primary care-based lifestyle and dietetic interventions, Tier 3 comprises specialist weight management services inclusive of multi-disciplinary team-based medical weight management, and Tier 4 covers specialised complex obesity services including bariatric surgery—which is subject to special commissioning. The National Institute for Health and Care Excellence (NICE) endorses bariatric surgery as a treatment option, in brief, for people with a BMI ≥ 40.0 kg/m^2^, or with a BMI of 35.0–39.9 kg/m^2^ with a significant obesity-related comorbidity, or with a BMI of 30.0–34.9 kg/m^2^ with T2D of less than 10 years’ duration [16].

Bariatric surgery is well established as a highly effective intervention in chronic severe obesity, leading to the resolution of comorbidities associated with obesity such as T2D, hypertension, hyperlipidaemia, OSA, and microvascular disease, as well as a reduction in mortality in patients over the long term [8,17,18]. The prospective Swedish Obese Subjects study concluded that bariatric surgery was superior to conventional treatment in terms of reduction in mortality and beneficial effects on comorbidities [19]. A recent study has reaffirmed that metabolic surgery is more effective than conventional medical therapy in the long-term control of T2D [20].

Bariatric surgical operations have evolved over the years with sleeve gastrectomy and Roux-en-Y gastric bypass (RYGB) currently being the dominant procedures, together accounting for nearly 90% of all operations performed worldwide [21]. Weight loss plays an important role in the reversibility of metabolic risk factors, with bariatric surgery resulting in high remission rates [22]. Whether patients with metabolic syndrome achieve weight loss that is comparable to those without metabolic syndrome and achieve long-term remission of metabolic factors is less clear. Although bariatric surgical outcomes of patients with metabolic syndrome postoperatively are widely reported in the literature, there is a paucity of comparative cohort studies looking at a non-metabolic syndrome control group and their parallel outcomes. Therefore, our study aimed to add to this gap in knowledge by examining the postoperative outcomes of patients with obesity who had metabolic syndrome compared with those who did not have metabolic syndrome at baseline.

## 2. Methods

We performed a retrospective longitudinal cohort analysis of patients who had undergone bariatric surgery based on national guidelines [15,16] at a National Health Service (NHS) university teaching hospital in northwest England between October 2008 and November 2011. The bariatric surgical procedures were all performed laparoscopically and included RYGB or sleeve gastrectomy. RYGB involved the creation of a short 5 cm vertical gastric pouch along the lesser curvature of the stomach, guided by a 40-French orogastric tube, using staplers as described previously [23]. An antecolic antegastric Roux-en-Y gastrojejunostomy was designed with the bilioenteric limb measuring 100 cm and the alimentary limb measuring 100–150 cm, varying according to the patient’s BMI. A side-to-side jejunojejunostomy was constructed using an intracorporeal suturing technique, and an end-to-side gastrojejunostomy was constructed using the intracorporeally sutured technique over a 40-French orogastric tube. Non-absorbable sutures were used to routinely close jejunojejunal and Petersen’s mesenteric defects. Laparoscopic sleeve gastrectomy involved the formation of a vertical gastric sleeve guided by a 40-French orogastric tube starting 4–6 cm from the pylorus and ending approximately 1 cm lateral to the angle of His, using staplers.

The World Health Organization (WHO) criteria for metabolic syndrome were adapted for the definition of metabolic syndrome in this study (Table 1) [24].

Patients were identified from an existing bariatric surgery database and clinical information was extracted from electronic patient records [25]. After excluding patients with insufficient baseline information to categorise the presence or absence of metabolic syndrome; 333 patients were included in the study and categorised into *Group A* (metabolic syndrome absent) and *Group B* (metabolic syndrome present). The electronic patient record was used to collect preoperative baseline and postoperative follow-up data at median 4; 12; 24; 36; 48; and 60 months; including demographic information; weight; height; BMI; percent total weight loss (%TWL; computed as: [weight loss ÷ initial weight] × 100); glycated haemoglobin (HbA1c); blood pressure (BP); total cholesterol; high-density lipoprotein (HDL) cholesterol; non-HDL cholesterol (the difference of total cholesterol and HDL cholesterol) and triglycerides.

### Statistical Analysis

We carried out a descriptive statistical analysis of demographic data, utilising parametric and non-parametric tests as appropriate. Descriptive analyses were reported as mean ± standard deviation (SD) for normally distributed data or median ± interquartile range (IQR) for non-normally distributed data. Categorical variables were analysed by the Fisher exact test and comparative analyses between groups with the Student *t* test. To cope with missing values, frequencies were reported as valid percentages. Significance level was set at *p* < 0.05. IBM SPSS Statistics 25.0 (IBM Corp., Armonk, NY, USA) was used for analysis.

## 3. Results

Of the 333 patients included in this study, 239 (71.8%) were women and 94 (28.2%) were men (Table 2). The bariatric surgical operations included 280 RYGBs (84.1%) and 53 sleeve gastrectomies (15.9%), with no 30-day mortality in the cohort. There were 133 patients without metabolic syndrome (*Group A*) and 200 with metabolic syndrome (*Group B*). There were no significant differences in baseline weight or BMI between the groups (Table 2). However, patients in *Group B* were older, with a greater proportion of men and those with T2D, hypertension, dyslipidaemia, and OSA.

### 3.1. Weight Loss Outcomes

**Figure 1:**
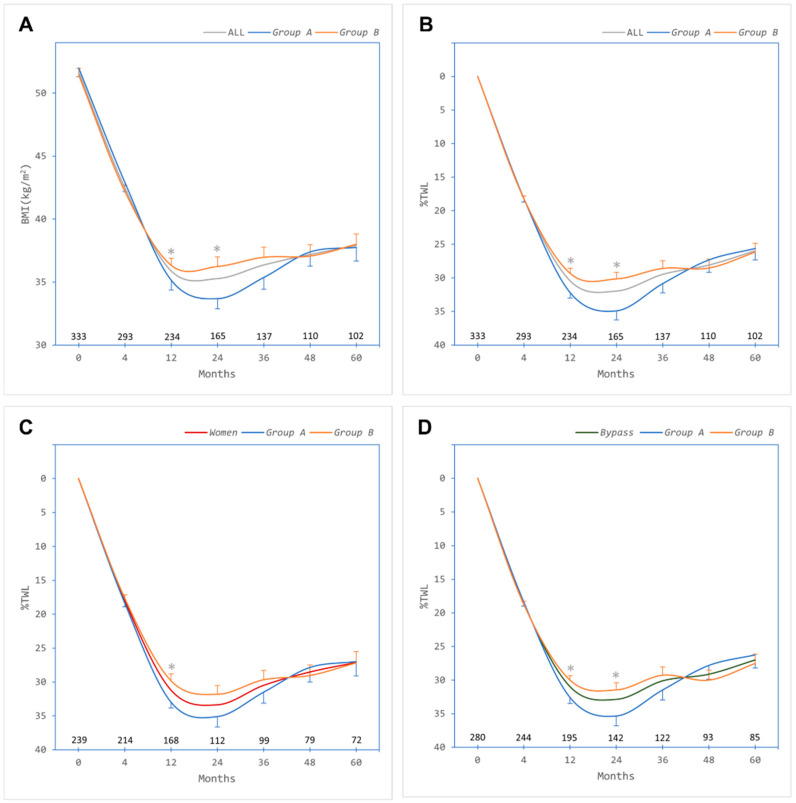
Weight loss outcomes after bariatric surgery over 60 months of follow-up. (**A**) Body mass index (BMI) in all patients (ALL, grey trace) and categorised by absence of metabolic syndrome (*Group A*, blue trace) or presence of metabolic syndrome (*Group B*, orange trace). (**B**) Percent total weight loss (%TWL) in all patients (ALL, grey trace) and categorised by absence of metabolic syndrome (*Group A*, blue trace) or presence of metabolic syndrome (*Group B*, orange trace). (**C**) %TWL in women (*Women*, red trace), who constituted 72% of the total cohort, categorised by absence of metabolic syndrome (*Group A*, blue trace) or presence of metabolic syndrome (*Group B*, orange trace). (**D**) %TWL in patients who underwent Roux-en-Y gastric bypass (*Bypass*, green trace), who constituted 84% of the total cohort, categorised by absence of metabolic syndrome (*Group A*, blue trace) or presence of metabolic syndrome (*Group B*, orange trace). Numbers of patients contributing data at each timepoint are depicted above the *x*-axis. Error bars, standard errors of the means. * Significant difference at timepoint between *Group A* and *Group B* (independent samples Student *t* test).

Overall baseline mean ± SD weight and BMI were 142.6 ± 27.1 kg and 51.7 ± 7.5 kg/m^2^, respectively. There was a significant reduction in BMI overall and in both groups (Figure 1A). Maximum %TWL at 24 months after bariatric surgery was 31.9% overall and 34.9% in *Group A* vs. 30.2% in *Group B* (*p* = 0.006); however, there were no significant differences in %TWL at 36, 48, and 60 months between groups (Figure 1B). Amongst women (who constituted 72% of the total cohort), there was no significant difference between *Group A* and *Group B* (Figure 1C). Similarly, amongst patients who had undergone RYGB (who constituted 84% of the total cohort), there was no significant difference in %TWL between *Group A* and *Group B* (Figure 1D).

### 3.2. Glycaemic Outcomes

**Figure 2:**
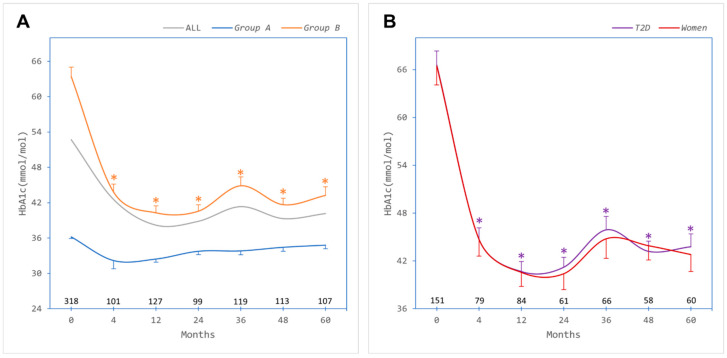
Glycaemic outcomes after bariatric surgery. (**A**) Glycated haemoglobin (HbA1c) in all patients (ALL, grey trace) and categorised by absence of metabolic syndrome (*Group A*, blue trace) or presence of metabolic syndrome (*Group B*, orange trace). (**B**) HbA1c in patients with type 2 diabetes (*T2D*, purple trace) and in females (*Women*, red trace), who constituted 62% of the people with T2D. Numbers of patients contributing data at each timepoint are depicted above the *x*-axis. Error bars, standard errors of the means. * Significant difference at timepoint compared with baseline (paired samples Student *t* test).

Baseline mean ± SD HbA1c was 36.2 ± 3.0 mmol/mol in *Group A* and 63.4 ± 22.1 mmol/mol in *Group B*. Although both groups achieved a reduction in HbA1c, it was significant in *Group B* (36.4% reduction at 12 months), and this trend was maintained over the follow-up period of 60 months (Figure 2A). In the 158 patients with T2D, baseline HbA1c was 66.6 ± 21.9 mmol/mol and reduced significantly after bariatric surgery (38.9% reduction at 12 months), including amongst women (who constituted 62% of the people with T2D) (Figure 2B).

### 3.3. Lipids and Blood Pressure

**Figure 3:**
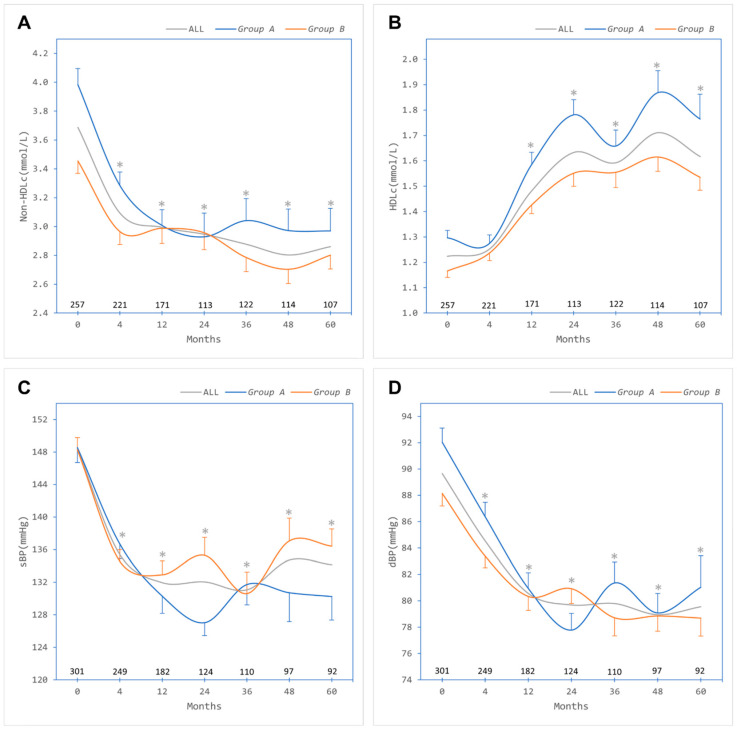
Lipid and blood pressure outcomes after bariatric surgery in all patients (ALL, grey trace) and categorised by absence of metabolic syndrome (*Group A*, blue trace) or presence of metabolic syndrome (*Group B*, orange trace). (**A**) Non-high-density lipoprotein cholesterol (Non-HDLc). (**B**) High-density lipoprotein cholesterol (HDLc). (**C**) Systolic blood pressure (sBP). (**D**) Diastolic blood pressure (dBP). Numbers of patients contributing data at each timepoint are depicted above the *x*-axis. Error bars, standard errors of the means. * Significant difference at timepoint compared to baseline (paired samples Student *t* test).

Overall baseline mean ± SD non-HDL cholesterol was 3.7 ± 1.1 mmol/L and decreased significantly in both groups following bariatric surgery (Figure 3A), with a simultaneous significant increase in HDL cholesterol (Figure 3B). Baseline systolic BP was 148.4 ± 20.1 mmHg and reduced significantly following bariatric surgery in both groups (Figure 3C). Similarly, diastolic BP reduced significantly from 89.7 ± 12.7 mmHg at baseline in both groups (Figure 3D).

## 4. Discussion

We evaluated longitudinal weight loss and metabolic outcomes up to five years after bariatric surgery in people with or without metabolic syndrome at baseline. We found significant and sustained weight loss and improvements in blood pressure and lipid profiles in both groups, with no significant differences between groups. Glycaemic control also improved significantly in the people with metabolic syndrome.

We found that maximum average weight reduction occurred between 12 and 24 months. This is similarly reported by the Swedish Obese Subjects study which found maximum weight loss was achieved between one and two years [19]. Other studies have reported 12 months for significant weight loss and maximum weight loss between 12 months and 18 months [8,26]. It is not unknown for patients to experience weight regain past these periods, as also seen in our study.

A 13-cohort study by the Metabolic Syndrome and Arteries Research Consortium comprising 34,821 participants from Europe and the US found a metabolic syndrome prevalence of 24% in the general population [27]. With the rising prevalence of obesity and related comorbidities, this number is expected to rise. The 60% prevalence of metabolic syndrome in our bariatric surgical cohort, which is much higher than the prevalence of metabolic syndrome in the general population, is reflective of referral and commissioning criteria for bariatric surgery that discriminate in favour of people with severe obesity who also have metabolic comorbidities. This is comparable to the 65% rate of metabolic syndrome in a recent Portuguese study [26].

It has long been recognised that metabolic bariatric surgery is the only intervention to consistently yield clinically effective short-term and long-term weight loss and metabolic outcomes in people with severe obesity [14,28]. These include improvement and remission of obesity-related comorbid metabolic conditions, particularly T2D, and improvement in quality of life and life expectancy [29]. Although most patients undergoing bariatric surgery at our centre were women, metabolic syndrome was more prevalent in men. It has previously been postulated that women are more likely to be willing to come forward and receive surgical treatment as compared with men [22]. Our group has previously shown that there were no significant differences in weight loss and metabolic outcomes between men and women matched for six key baseline characteristics including age, BMI, type of bariatric procedure, presence of T2D, insulin treatment, and OSA treated with continuous positive airway pressure [30]. Effective strategies for improving uptake of bariatric surgery in all eligible patients, including men and marginalised groups, are needed.

There may, however, be sex-specific differences in body composition following metabolic bariatric surgery that are associated with remission of metabolic syndrome. A recent study has found that reduction in visceral adipose tissue might be related to reversibility of metabolic syndrome in males, whilst reduced lean body mass may be associated with non-remission in females [31].

It is well reported in the literature that bariatric surgery does cause a significant reversal of dysmetabolism, and this is largely attributed to the considerable weight loss from bariatric surgery [8,22,32]. Despite this, it is interesting that some authors have described a lack of dependency on weight loss for the reversal of comorbidities such as hypertension, T2D, and dyslipidaemia in their cohort [33]. This could perhaps be attributed to the biliopancreatic diversion and Roux gastroenteroanastamosis technique that was used in their cohort.

Although the pathogenesis of metabolic syndrome and its links to obesity are not fully understood, it is thought that adipocytokines such as adiponectin, tumour necrosis factor-α (TNF-α), and resistin play a key role [34,35]. Non-esterified fatty acid metabolism has been observed to contribute to insulin resistance in those with excess visceral fat. Visceral obesity is also associated with a pro-inflammatory state including the release of pro-inflammatory cytokines and macrophage infiltration into adipose tissues. C-reactive protein is also raised in patients with obesity and predicts a risk of myocardial infarctions. This pro-inflammatory state could be instrumental to the insulin resistance, dysglycaemia, dyslipidaemia and hypertension that is seen in these patients.

Another hypothesis that links metabolic syndrome with visceral obesity suggests that subcutaneous fat is unable to take in the excess energy from a caloric surplus [35]. This reduced ability of the subcutaneous adipose tissue to take in excess fat leads to the accumulation of fat in other undesirable places such as the liver, the heart, and skeletal muscle. This has an effect of increased insulin resistance leading to the development of T2D. Whereas hyperinsulinaemia was initially thought to be the cause of the metabolic derangements, it is now well recognised that insulin resistance is the key factor in the development of metabolic syndrome [36]. Visceral obesity results in a chronic inflammatory state responsible for the production of cytokines such as interleukin-6, leptin, resistin, and TNF-α. These abnormally present cytokines inhibit insulin signalling in hepatocytes, resulting in impaired suppression of gluconeogenesis causing hyperglycaemia, which then leads to an increased production of very low-density lipoprotein and C-reactive protein levels [35]. These abnormalities have been shown to promote atherosclerosis, leading to the eventual cardiovascular complications seen in patients with advanced metabolic syndrome. Thus, the significant reduction in glycaemic exposure seen in our cohort of patients from both groups, particularly the 36% reduction in HbA1c at 12 months after bariatric surgery in people with metabolic syndrome, is encouraging. This is supported by meta-analysis from Buchwald et al., who reported that insulin levels declined significantly postoperatively, as did HbA1c and fasting glucose levels [37].

Both groups in our study had high mean systolic and diastolic blood pressures at baseline with a significant decrease by four months. Obesity is associated with hypertension through various mechanisms, including chronic vascular inflammation, oxidative stress, activation of the renin–angiotensin–aldosterone system (RAAS), and sympathetic overdrive [38]. Weight loss and consequent improvements in obesity-related dysmetabolic and chronic inflammatory states eventually lead to lowering of blood pressure. Generally speaking, a decrease in body weight of 1% leads to a 1 mmHg decrease in systolic and a 2 mmHg decrease in diastolic blood pressure [8]. However, blood pressure reduction becomes evident within a week after bariatric surgery [39], attributable to neurohormonal mechanisms—such as an increase in incretin levels—and only later to weight reduction [40]. Reduction of renal sinus fat after bariatric surgery has also been associated with the lowering of blood pressure by alleviating pressure effects on the renal vein and decreasing activation of RAAS [41].

Metabolic bariatric surgery has also found a role in the treatment of severe obesity in adolescents and young persons [42]. One study has shown that adolescents and adults who underwent gastric bypass had significant, similar, and sustained weight reduction up to five years after surgery, with higher rates of remission of T2D and hypertension in adolescents than in adults [43]. However, little is known about the relationship between metabolic factors and weight loss success in adolescents undergoing bariatric surgery. A recent study has found that raised systolic blood pressure and HbA1c in adolescents with morbid obesity may reflect a target population more likely to achieve successful weight loss [44].

### Limitations

A strength of our pragmatic, real-world observational study is long-term prospective data collection, despite limitations of retrospective design and data attrition due to patients lost to follow-up. Whilst we did not record ethnic demographics, our catchment population comprises mostly White British ethnicity. It was beyond the scope of the study to determine data on personalised dose adjustments of medications and patients’ adherence to treatment, which may have influenced metabolic parameters of interest during follow-up. There was clear evidence of improvements in measurable endpoints including weight, HbA1c, blood pressure, and lipid parameters; therefore, we did not seek to reclassify metabolic syndrome status at follow-up timepoints.

## 5. Conclusions

Metabolic syndrome is highly prevalent in patients referred for bariatric surgery. We report that bariatric surgery is equally effective for weight loss in patients with or without metabolic syndrome. Postoperatively, metabolic factors such as blood pressure, lipid profile, and glycaemic control improved in both study groups. Further research could investigate sustained long-term clinical effectiveness and cost-effectiveness outcomes of bariatric surgery versus medical therapies in patients with obesity at a high risk of metabolic syndrome.

## Figures and Tables

**Table 1:** Definition of metabolic syndrome.

Modified WHO Criteria for Definition of Metabolic Syndrome	
	Notes
Essential criteria	
Obesity with BMI ≥ 30.0 kg/m^2^	All patients fulfilled the BMI requisite; waist-to-hip ratios were not consistently available and, therefore, not included
Known T2D or evidence of insulin resistance (HbA1c ≥ 42 mmol/mol)	Blood glucose values were not consistently available and, therefore, HbA1c was used
AND one or more of the following criteria:	
Known dyslipidaemia or raised TG (≥1.7 mmol/L) or low HDLc (<0.9 mmol/L in men, <1.0 mmol/L in women)	
Known hypertension or systolic BP ≥ 140 mmHg or diastolic BP ≥ 90 mmHg	
	Urinary albumin excretion measurements were not consistently available and, therefore, not included

The World Health Organization (WHO) criteria for the definition of metabolic syndrome were adapted for use in this study. BMI, body mass index. T2D, type 2 diabetes. TG, triglycerides. HDLc, high-density lipoprotein cholesterol. BP, blood pressure.

**Table 2:** Baseline characteristics of all participants categorised by absence (*Group A*) or presence of metabolic syndrome (*Group B*).

	All (n = 333)	*Group A* (n = 133)	*Group B* (n = 200)	*p* *
Women:Men	239 (71.8%):94 (28.2%)	110 (82.7%):23 (17.3%)	129 (64.5%):71 (35.5%)	<0.001
Bypass:Sleeve	280 (84.1%):53 (15.9%)	113 (85.0%):20 (15.0%)	167 (83.5%):33 (16.5%)	ns
Age (years)	46.4 (10.7)	41.3 (10.1)	49.7 (9.7)	<0.001
Weight (kg)	142.6 (27.1)	142.1 (29.1)	142.9 (25.7)	ns
BMI (kg/m^2^)	51.7 (7.5)	52.0 (7.9)	51.4 (7.2)	ns
Type 2 diabetes	158 (47.4%)	0 (0.0%)	158 (79.0%)	<0.001
Hypertension	170 (51.1%)	36 (27.1%)	134 (67.0%)	<0.001
Hyperlipidaemia	85 (25.5%)	18 (13.5%)	67 (33.5%)	<0.001
OSA (CPAP)	107 (32.1%)	31 (23.3%)	76 (38.0%)	<0.006

Values are count (percent) or mean (standard deviation). * *Group A* vs. *Group B* (Fisher exact test for 2 × 2 contingency tables; Student *t* test for continuous variables). ns, non-significant. BMI, body mass index. OSA (CPAP), obstructive sleep apnoea treated with continuous positive airway pressure.

## Data Availability

The data presented in this study are not publicly available due to clinical service evaluation restrictions.
